# Perioperative Anesthetic Management in Pediatric Scoliosis Surgery: A Narrative Review with Focus on Neuromuscular Disorders

**DOI:** 10.3390/children12111481

**Published:** 2025-11-02

**Authors:** Barbora Nedomová, Boris Liščák, Soňa Urbanová, Štefan Pavlík, Rudolf Riedel, Vlasta Dostálová

**Affiliations:** 1 Department of Pediatric Anesthesiology and Intensive Medicine, Faculty of Medicine, Comenius University and National Institute of Children’s Diseases, 833 40 Bratislava, Slovakia; 2 Department of Orthopedics, Faculty of Medicine, Comenius University and National Institute of Children’s Diseases, 833 40 Bratislava, Slovakia; 3 Department of Radiology, National Institute of Children’s Diseases, 833 40 Bratislava, Slovakia; 4 Department of Anesthesiology and Intensive Care Medicine, Charles University, Faculty of Medicine in Hradec Kralove, University Hospital Hradec Kralove, Sokolska 581, 500 05 Hradec Kralove, Czech Republic

**Keywords:** pediatric anesthesia, scoliosis, neuromuscular disorders, total intravenous anesthesia, perioperative management, blood conservation, ERAS protocol

## Abstract

**Highlights:**

**What are the main findings?**

- Individualized anesthetic planning with total intravenous anesthesia and blood conservation is essential in neuromuscular scoliosis surgery.
- Implementing Enhanced Recovery After Surgery principles facilitates faster recovery and reduces complications in this high-risk population.

**What is the implication of the main finding?**

- A multidisciplinary, evidence-based approach improves perioperative outcomes.
- Standardized protocols enhance safety and recovery in pediatric neuromuscular scoliosis surgery.

**Abstract:**

Background/Objectives: Scoliosis surgery in pediatric patients, particularly those with neuromuscular disorders, is associated with increased perioperative risk due to respiratory insufficiency, cardiovascular comorbidities, and nutritional deficiencies. This review aims to summarize current evidence-based approaches to anesthetic management in this vulnerable population. Methods: A comprehensive literature review was conducted focusing on anesthetic strategies and multidisciplinary protocols used in the perioperative care of children with neuromuscular conditions undergoing scoliosis surgery. Emphasis was placed on intraoperative neurophysiological monitoring (IONM), blood conservation techniques, and Enhanced Recovery After Surgery (ERAS) principles. Results: Key management strategies include individualized preoperative risk assessment, use of total intravenous anesthesia (TIVA) to preserve IONM signal integrity, and the implementation of blood conservation methods such as antifibrinolytic therapy and intraoperative cell salvage. Additional perioperative considerations include maintaining normothermia, careful positioning, and multimodal analgesia. Postoperative care should incorporate structured respiratory support and early mobilization within the ERAS pathway to promote recovery and reduce complications. Conclusions: The perioperative care of pediatric patients with neuromuscular scoliosis undergoing spinal surgery requires a multidisciplinary and individualized anesthetic approach. Adherence to evidence-based protocols, including TIVA, blood management strategies, and ERAS principles, is essential for minimizing perioperative complications and improving outcomes in this high-risk group.

## 1. Introduction

**Figure 1:**
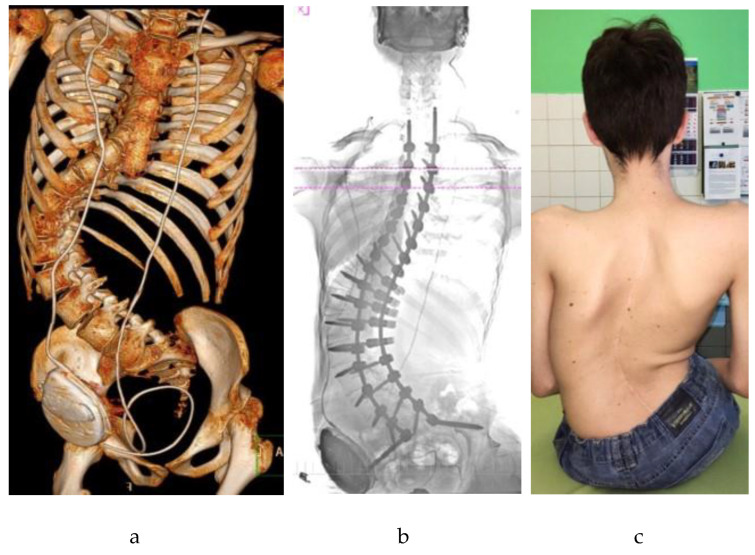
An adolescent patient with neuromuscular scoliosis secondary to cerebral palsy and hydrocephalus, following posterior spinal fusion with pelvic fixation: (**a**) Three-dimensional computed tomography (CT) reconstruction showing a severe scoliotic deformity with rotational and translational components, corrected by posterior instrumentation; (**b**) Anteroposterior radiograph illustrating long-segment posterior spinal fusion extending to the pelvis; (**c**) Postoperative clinical image in the seated position showing a midline surgical scar and improved spinal alignment.

**Figure 2:**
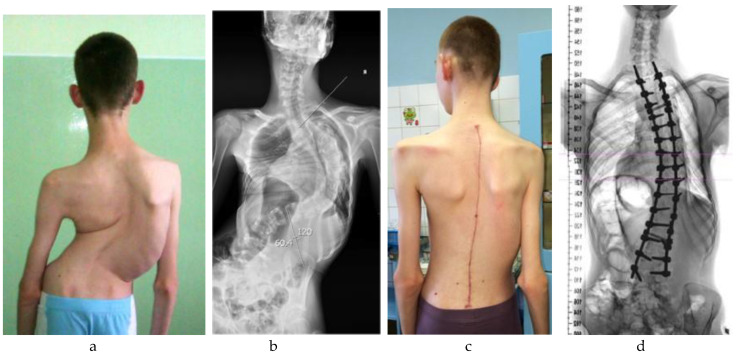
An adolescent patient with neuromuscular scoliosis secondary to cerebral palsy. In contrast to the case presented in Figure 1, this patient retained ambulatory function and did not require pelvic fixation: (**a**) Preoperative clinical image demonstrating moderate coronal imbalance with visible scapular asymmetry; (**b**) Preoperative standing anteroposterior radiograph showing a structural thoracolumbar scoliosis without pelvic obliquity; (**c**) Postoperative clinical image showing improved trunk alignment and a well-healed midline incision; (**d**) Postoperative radiograph demonstrating correction of the scoliotic curve with segmental posterior instrumentation terminating above the pelvis.

The surgical correction of scoliosis in pediatric patients with neuromuscular disorders remains one of the most complex challenges in pediatric anesthesiology [1,2]. Neuromuscular scoliosis (NMS), defined as a structural spinal deformity secondary to an underlying neuromuscular condition (e.g., cerebral palsy [CP; see Figure 1 and Figure 2], spinal muscular atrophy [SMA], Duchenne muscular dystrophy [DMD]), is frequently encountered in clinical practice [2,3,4]. NMS arises from progressive muscular weakness and impaired postural control, often leading to severe three-dimensional deformities and thoracic insufficiency [2,3,5]. Epidemiologically, scoliosis is common across neuromuscular conditions, with prevalence increasing alongside functional severity and age. In CP, population-based pediatric cohorts show that at 10 years ≈1% (Gross Motor Function Classification System [GMFCS] I–II), ≈5% (GMFCS III), ≈10% (GMFCS IV), and ≈30% (GMFCS V) have scoliosis; by 20 years, ≈75% of those in GMFCS V reach a Cobb angle ≥ 40°, whereas none in GMFCS I do so [6,7]. In SMA, pediatric series report scoliosis in ≈90% of type I, 80–90% of type II, and ≈50% of type III patients [8,9]. In DMD, curves typically emerge after loss of ambulation. However, contemporary long-term corticosteroid therapy—by prolonging ambulation—has been associated with a lower incidence and delayed onset compared with earlier cohorts [10]. These differences underscore the perioperative burden and the importance of condition-specific anesthetic planning.

Children with NMS frequently present with multisystem comorbidities—restrictive pulmonary disease with ineffective airway clearance and recurrent infections, swallowing dysfunction with gastroesophageal reflux, and malnutrition—which collectively elevate perioperative risk and mandate individualized anesthetic strategies [11]. Additional complexity arises from altered pharmacokinetics and an increased risk of respiratory compromise during long posterior fusions [12]. Furthermore, anesthetic maintenance must preserve the integrity of intraoperative neurophysiological monitoring (IONM), a topic expanded in the intraoperative section [13].

This review synthesizes perioperative anesthetic principles specific to NMS with a pragmatic, clinician-focused emphasis on risk stratification, airway and respiratory management, IONM-compatible anesthesia, blood conservation, and postoperative care within multidisciplinary Enhanced Recovery After Surgery (ERAS) pathways [14]. A narrative review format was adopted because the pediatric evidence base spans heterogeneous sources—society guidelines and consensus statements (e.g., Scoliosis Research Society, Pediatric Orthopaedic Society of North America, and anesthesia societies), pediatric cohort studies, technique-oriented reports, and neurophysiology recommendations—with substantial variability in populations, interventions, and outcomes that preclude meaningful meta-analysis [11,12,13,14]. A narrative synthesis enables integration of guideline-level recommendations with pragmatic perioperative protocols most relevant to anesthesiologists managing NMS. Methods detail our search process and evidence weighting.

## 2. Materials and Methods

### 2.1. Design and Scope

This narrative review synthesizes current evidence on perioperative anesthetic management for pediatric spinal deformity surgery, focusing on NMS.

### 2.2. Data Sources and Search Strategy

The primary database was PubMed/MEDLINE, queried from January 2010 through 30 August 2024. Earlier landmark studies were included when foundational to current practice. Reference lists of key articles and webpages of relevant societies (e.g., Scoliosis Research Society [SRS], Pediatric Orthopaedic Society of North America [POSNA], and major anesthesia societies) were hand-searched. Search strings combined controlled vocabulary (Medical Subject Headings [MeSH]) and free-text keywords reflecting the perioperative pathway (e.g., neuromuscular scoliosis, pediatric/children, anesthesia/anesthetic management, airway, ventilation, total intravenous anesthesia, intraoperative neuromonitoring, tranexamic acid/blood conservation, ERAS). Terms on surgical planning—computed tomography (CT) for pedicle-screw or osteotomy planning, navigation/robotic assistance, and halo-gravity traction (HGT)—were added to capture evidence relevant to Section 4. The search was last updated on 30 August 2024.

### 2.3. Eligibility Criteria

Eligible studies included peer-reviewed, primarily English-language publications directly applicable to pediatric perioperative care in NMS: guidelines, consensus statements, clinical trials, case series, cohort studies, and systematic or narrative reviews. Two Slovak publications were retained for their original data on spinal decompression techniques. Excluded materials were non-peer-reviewed works, single-patient case reports, conference abstracts without full text, and adult-only series without pediatric relevance. Where pediatric data were limited, high-quality adolescent or mixed-population studies were included when perioperative pathways were compatible.

### 2.4. Study Selection and Yield

Titles and abstracts, followed by full texts, were independently screened by two authors, with disagreements resolved by consensus. Across all PubMed queries, 653 records were identified. After de-duplication across query modules, 574 unique records were screened at the title and abstract level; a total of 89 publications, including those identified through hand-searching of society webpages and citation lists, were retained for qualitative synthesis. This approach en-sures quantitative transparency while remaining consistent with the narrative design of the review.

### 2.5. Quality Considerations and Evidence Weighting

Given heterogeneity in study designs, populations (e.g., CP, SMA, DMD, MMC), interventions, and outcomes, no single formal risk-of-bias instrument was applied. Instead, a prespecified qualitative hierarchy was used:

(i) Society guidance (SRS, POSNA, anesthesia societies) and multicenter/prospective pediatric cohorts were weighted more heavily than single-center descriptive series;
(ii) Age- and diagnosis-specific applicability (pediatric NMS over adolescent idiopathic scoliosis (AIS)/adult data) was prioritized;
(iii) Recency, sample size, and presence of comparators informed interpretation when recommendations conflicted.

Use of adolescent or mixed-population evidence is explicitly indicated where pediatric NMS data were lacking.

### 2.6. Data Extraction and Synthesis

From included sources, we abstracted study scope; population and diagnosis; anesthetic techniques (including IONM compatibility); ventilation strategies for restrictive disease; blood-conservation measures (including tranexamic acid protocols); perioperative pathways (prehabilitation, extubation or non-invasive ventilation (NIV) criteria, ERAS elements); and complication incidences. Findings were synthesized thematically by perioperative phase: preoperative assessment and optimization, intraoperative management, perioperative complications, and postoperative care.

### 2.7. Ethics

No original human or animal data were generated for this review; institutional ethics approval was not required.

## 3. Preoperative Assessment and Optimization

The therapeutic approach to spinal deformities in pediatric patients relies on accurate classification that considers age, underlying etiology, and the characteristics and extent of the spinal curvature. This assessment is essential for selecting a tailored treatment strategy. The primary goals of surgical intervention are spinal stabilization, prevention of curve progression, and maximal safe deformity correction, accompanied by pain reduction, improved functional capacity, and enhanced quality of life for patients and caregivers.

Preoperative preparation for scoliosis surgery in children is a complex, multiphase process aimed at creating optimal conditions for safe surgery under general anesthesia. Its main objectives are to conduct a thorough perioperative risk assessment, optimize the child’s clinical condition, formulate an individualized anesthetic plan, and define the scope of required intra- and postoperative monitoring. Given the physiological demands of spinal surgery on multiple organ systems, the extent of preoperative evaluation must be adapted to age, diagnosis, and comorbidities, with early involvement of a multidisciplinary team (anesthesiology, orthopedics, neurology, pulmonology, cardiology, rehabilitation, nutrition) and clear care pathways [15].

### 3.1. Anesthetic Pre-Assessment

Evaluation should encompass the child’s overall medical condition and potential difficulties in airway management. Baseline laboratory tests typically comprise a complete blood count, coagulation profile, and basic metabolic panel; additional testing is guided by comorbidities and medications (e.g., antiepileptics, intrathecal baclofen). Depending on clinical findings, further specialist consultations—neurology, pulmonology, and cardiology—may be warranted [15].

### 3.2. Pulmonary Evaluation

Assessment is tailored to age, clinical status, and cooperation. Recommended investigations include overnight oxygen saturation monitoring and/or capnography to screen for nocturnal hypoventilation, with post-sleep arterial or capillary blood gas analysis when feasible. Polysomnography provides details on sleep-related breathing disturbances. Cooperative children should undergo spirometry (forced vital capacity [FVC], forced expiratory volume in 1 s [FEV_1_]); in neuromuscular disorders, evaluation of cough strength and maximal inspiratory/expiratory pressures (MIP/MEP) is advised. In severe thoracic deformity or abnormal pulmonary function, chest CT can be considered to evaluate airway compression and parenchymal disease [15,16].

### 3.3. Respiratory Optimization

A proactive strategy is recommended, emphasizing early airway-clearance techniques (e.g., oscillatory devices, mechanical insufflation–exsufflation), respiratory physiotherapy, and timely initiation or optimization of NIV when indicated. These interventions reduce perioperative pulmonary complications and facilitate recovery; planned postoperative NIV and routine cough-assist should be considered in high-risk children [17,18,19].

### 3.4. Cardiac Evaluation

Cardiac assessment is essential when cardiovascular involvement is suspected or confirmed. Electrocardiography and transthoracic echocardiography are recommended—particularly for disorders associated with cardiomyopathy or conduction abnormalities (e.g., DMD)—to guide hemodynamic targets, rhythm surveillance, and anesthetic planning [20,21].

### 3.5. Nutritional Optimization and Aspiration Risk

Malnutrition is common and independently associated with adverse outcomes in neuromuscular scoliosis. Early dietetic assessment, caloric/protein optimization, and correction of micronutrient deficiencies (e.g., iron, vitamin D) are recommended. In selected patients with severe dysphagia or reflux—especially in SMA and DMD—prehabilitation may include establishing a reliable enteral route (e.g., gastrostomy) weeks to months before surgery to stabilize weight and reduce aspiration risk. Aspiration mitigation involves reflux management when present, adherence to pediatric fasting guidance, individualized modified rapid-sequence induction in high-risk airways, and explicit airway-protection strategies at extubation (e.g., cuff-leak assessment, optimal positioning, early NIV) [16,22].

### 3.6. Psychological Preparation and Family Communication

Structured preoperative counseling reduces anxiety and improves adherence to postoperative respiratory therapy and mobilization. Discussions should set expectations for the ICU course, pain control, the potential need for NIV and cough-assist, and escalation pathways; they should also—sensitively—acknowledge the possibility of serious complications, including neurological injury and mortality, to support informed consent and shared decision-making [23].

### 3.7. Blood Management Planning

Preoperative blood-conservation planning should include protocolized antifibrinolysis (tranexamic acid, TXA) [24,25,26,27], temperature management, and intraoperative cell salvage when available. Cross-matching should reflect the estimated blood volume and anticipated loss based on curve severity and planned fusion length. Given the potential for major hemorrhage in long posterior fusions, institutions should maintain explicit massive transfusion protocol (MTP) readiness with predefined activation criteria, component therapy guidance, and point-of-care coagulation testing where available.

Transfusion thresholds should be defined within institutional protocols and individualized to each child’s physiologic status and comorbidities, in coordination with postoperative monitoring plans.

Centers are encouraged to adopt a standardized TXA protocol consistent with local experience and safety monitoring. Evidence supports TXA effectiveness across pediatric scoliosis, with several cohorts showing greater blood-sparing at higher doses and randomized/meta-analytic data in AIS confirming benefit at lower doses. Intraoperative details on coagulation management and dosing strategies are discussed in Section 5.5.

### 3.8. Venous Thromboembolism (VTE) Risk and Prophylaxis

Although pediatric VTE after elective spine surgery is rare in mixed pediatric cohorts, NMS may carry a relatively higher risk owing to immobility, prolonged procedures, central venous access, and multisystem comorbidity. Routine mechanical prophylaxis is recommended for all patients, and selective pharmacologic prophylaxis based on individual risk and institutional protocol, consistent with contemporary pediatric consensus [28].

### 3.9. Premedication and Anxiolysis

Premedication, an important perioperative planning component, should be tailored to the child‘s condition and comorbidities. Benzodiazepines are often avoided in children with neuromuscular scoliosis because of risks of respiratory depression, agitation, and postoperative cognitive effects; where anxiolysis is required, alternatives such as dexmedetomidine, clonidine, or low-dose ketamine may be considered, alongside non-pharmacologic strategies (child-life preparation, caregiver presence, distraction techniques) to minimize sedative burden [15,23].

## 4. Surgical Planning and Deformity Preparation

### 4.1. Preoperative Imaging and Navigation

Beyond pulmonary assessment, CT delineates pedicle morphology, vertebral rotation, and canal dimensions, thereby informing pedicle screw trajectories and osteotomy strategy in severe three-dimensional deformity and dysplastic pedicles. In experienced centers, CT-based navigation or robotic assistance can improve placement accuracy and reduce unplanned screw revision; the benefit must be balanced against radiation exposure and institutional resources. Where pediatric NMS-specific evidence is limited, evidence from adolescent cohorts is cautiously extrapolated due to shared posterior instrumentation steps [29,30,31].

### 4.2. Halo-Gravity Traction (HGT) for Severe, Rigid Curves

In children with large, rigid deformities and compromised respiratory mechanics, preoperative HGT can progressively reduce curve magnitude, improve ventilatory mechanics, and facilitate safer definitive correction with less need for aggressive osteotomies. Contemporary pediatric studies report meaningful coronal/sagittal correction with an acceptable safety profile when traction is escalated gradually with regular neurological checks, diligent pin-site care, and pressure-injury prevention. Courses beyond approximately 3 months seldom add benefit and increase care burden. Reported complications (generally infrequent) include pin-site infection/loosening, transient neuropraxia or cranial-nerve symptoms, neck discomfort, and dysphagia; standardized protocols and multidisciplinary oversight mitigate these risks [32,33,34,35,36,37,38].

### 4.3. Implications for Anesthesia and Team

Surgical planning that incorporates CT-guided trajectory/osteotomy maps and, when indicated, staged HGT should be tightly coordinated with anesthesia to: (i) anticipate airway management and positioning in traction; (ii) align hemodynamic goals with neuromonitoring requirements during gradual correction and definitive fusion; (iii) plan analgesia and skin/pressure protection during traction periods; and (iv) ensure clear intraoperative communication regarding traction adjustments and IONM milestones [29,30,31,32,33,34,35,36,37,38].

## 5. Anesthetic Techniques and Intraoperative Considerations

Scoliosis correction is among the most complex procedures in pediatric orthopedic practice, demanding meticulous anesthetic planning due to its technical demands and elevated perioperative risk. Key intraoperative considerations include optimal patient positioning and appropriate anesthetic techniques—particularly in the context of IONM—ventilatory management, blood conservation strategies, and early planning for postoperative intensive care [15,39,40,41].

Although NMS is often approached as a single entity, perioperative management should reflect condition-specific risks and priorities; the matrix below highlights high-impact differences relevant to anesthetic planning (Table 1) [15,39,41].

### 5.1. Intraoperative Respiratory and Positioning Management in NMS 

Given the reduced chest wall compliance and restrictive mechanics in NMS—exacerbated by prone positioning—we favor pressure-controlled ventilation (PCV) or pressure-regulated volume control (PRVC; also termed pressure-controlled ventilation–volume guaranteed [PCV–VG]), targeting low tidal volumes (~6–7 mL·kg^−^^1^ of predicted body weight [PBW]) with individualized PEEP (~5–8 cmH_2_O) to maintain oxygenation and dynamic compliance without impeding venous return. Gentle recruitment after turning prone, avoidance of nitrous oxide, maintenance of EtCO_2_ ~35–40 mmHg and normoxia, and limitation of peak and plateau pressures help protect the lung and preserve IONM signal quality [15,39].

**Figure 3:**
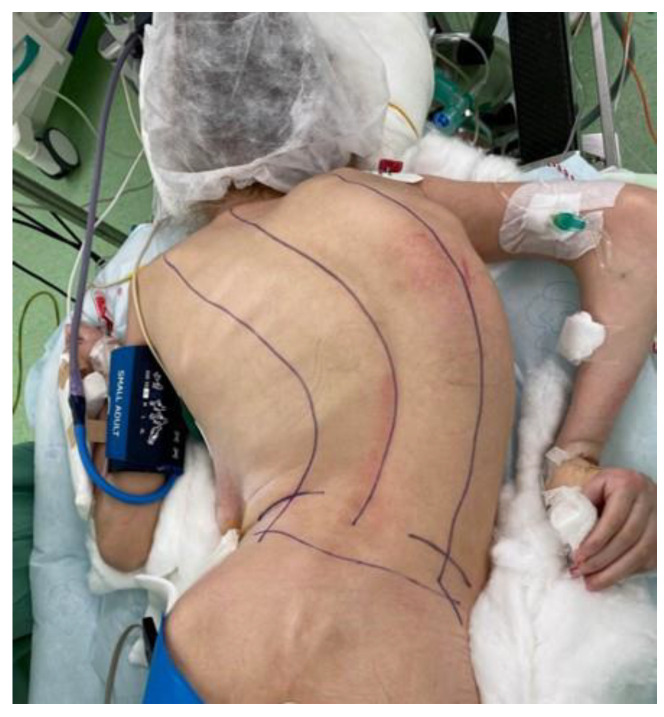
Intraoperative prone positioning in a pediatric patient undergoing scoliosis correction. Proper support of the thorax and abdomen and alignment of the spine are essential to minimize intra-abdominal pressure and avoid positioning-related complications.

Several factors may increase intra- and postoperative morbidity in these patients, including systemic comorbidities, major blood loss, risk of neurological injury, and the physiologic effects of prone positioning. When placing the patient in the prone position, adequately supporting the thorax and abdomen is essential to prevent increased intra-abdominal and intrathoracic pressures, which can impair venous return and cardiopulmonary interactions (Figure 3). Compression of the inferior vena cava may reduce preload and promote epidural venous engorgement, increasing the risk of bleeding from these highly vascular structures. Improper positioning may also reduce chest wall excursion, diminish pulmonary compliance, and decrease functional residual capacity [42].

The surgical and anesthetic teams are responsible for ensuring safe and physiologically favorable positioning. Before surgical draping, proper alignment must be confirmed; all pressure points protected; and measures taken to prevent nerve injuries to the brachial plexus, ulnar nerve, dorsal foot nerves, and lateral femoral cutaneous nerve. The endotracheal tube must be secured to avoid dislodgement. The eyes should be protected from direct pressure, with commercial face pillows or mirrors recommended for facial observation. A bite block should be inserted to protect oral structures during motor evoked potential (MEP) stimulation. If the face is not readily visible, indirect visualization using simple tools (e.g., smartphone camera) may be helpful [43].

Perioperative visual loss is a rare but potentially devastating complication of prone spinal surgery. Its estimated incidence is approximately 3.09 per 10,000 spine procedures [44,45]. Reported mechanisms include anterior and posterior ischemic optic neuropathy, central retinal artery occlusion, cortical blindness, and posterior reversible encephalopathy syndrome [45]. Although the precise pathophysiology in the prone position remains unclear, data from the American Society of Anesthesiologists Postoperative Visual Loss Registry identify prolonged anesthesia duration and considerable intraoperative blood loss as key contributing factors [46].

### 5.2. IONM

IONM markedly reduces the risk of neurological injury by assessing the functional integrity of neural pathways [47]. It enables early detection of irritation or injury to critical neural structures and facilitates real-time surgical adjustments. Historically, the Stagnara wake-up test was used intraoperatively to assess motor function in the lower limbs; however, this technique has largely been replaced with modern electrophysiological modalities [48,49].

Combined somatosensory-evoked potentials (SSEPs) and MEPs have become the standard of care for spinal deformity surgery. Successful IONM requires close collaboration among the surgeon, anesthesiologist, and neurophysiologist, with anesthetic management carefully tailored to preserve signal integrity.

TIVA using propofol and short-acting opioids (e.g., remifentanil) is preferred for IONM. Target-controlled infusion (TCI) systems are commonly used to ensure stable anesthetic depth. Typical ranges used with IONM include propofol 100–200 μg·kg^−^^1^·min^−^^1^ (or TCI ≈ 2–4 μg·mL^−^^1^) combined with remifentanil 0.05–0.2 μg·kg^−^^1^·min^−^^1^; adjunct ketamine 0.25–0.5 mg·kg^−^^1^ bolus then 0.1–0.3 mg·kg^−^^1^·h^−^^1^ can reduce propofol requirements without degrading MEPs; if dexmedetomidine is used, keep low infusions ≈ 0.2–0.5 μg·kg^−^^1^·h^−^^1^ and monitor for possible MEP attenuation [50]. In contrast, inhalational agents, nitrous oxide, and benzodiazepines depress MEP amplitudes and prolong latencies, and are generally avoided. Dexmedetomidine may also suppress MEPs, although its effects on SSEPs remain less clearly defined [51].

Non-depolarizing neuromuscular blockers may be administered during induction, but should be discontinued once IONM begins [50]. Physiological disturbances such as hypotension, hypovolemia, anemia, and hypothermia can impair monitoring quality and must be promptly addressed if changes in evoked potentials occur [50,52]. The structured multidisciplinary protocol for responding to IONM signal changes is presented in Table 2.

A well-coordinated intraoperative approach, encompassing optimized ventilation, maintenance of hemodynamic stability, effective thermoregulation, and management of anticipated blood loss, combined with precisely tailored anesthetic strategies and reliable neurophysiological monitoring, is key to ensuring surgical safety and preserving neurological function.

### 5.3. Perioperative Monitoring

The extent of perioperative vital sign monitoring during scoliosis surgery depends on the procedure’s type and duration, anticipated blood loss, and patient comorbidities. Standard monitoring during general anesthesia for spinal deformity surgery includes electrocardiography, heart rate, oxygen saturation (SpO_2_), invasive blood pressure, body temperature, ventilatory parameters, hourly urine output, and anesthesia depth. Near-infrared spectroscopy serves as a useful adjunct for continuous, noninvasive monitoring of regional cerebral oxygenation.

### 5.4. Perioperative Temperature Management

Intraoperative hypothermia is a major concern in pediatric scoliosis surgery, particularly in patients with neuromuscular disorders. Reported incidence in elective pediatric surgery ranges from 2.7% to 74%, depending on procedure type, age, and intraoperative conditions [53,54]. Children with neuromuscular conditions, severe spinal deformities, and low body mass index are especially vulnerable. Severe hypothermia, a 2.5 °C drop in core body temperature from baseline, can markedly impair IONM by reducing the quality of evoked potentials. Moreover, even mild hypothermia (1–2 °C decrease) has been shown to triple the risk of surgical site infections, prolong hospitalization by approximately 20%, and increase intraoperative blood loss [53].

Preventive measures should include preoperative warming, active intraoperative warming, use of warmed intravenous fluids, and continuous temperature monitoring via esophageal or nasopharyngeal probes. Recommended warming modalities include forced-air warming blankets, underbody warming pads, and fluid warming systems. Strict maintenance of normothermia is essential to reduce perioperative complications and supports better recovery.

### 5.5. Blood Loss in Scoliosis Surgery

**Figure 4:**
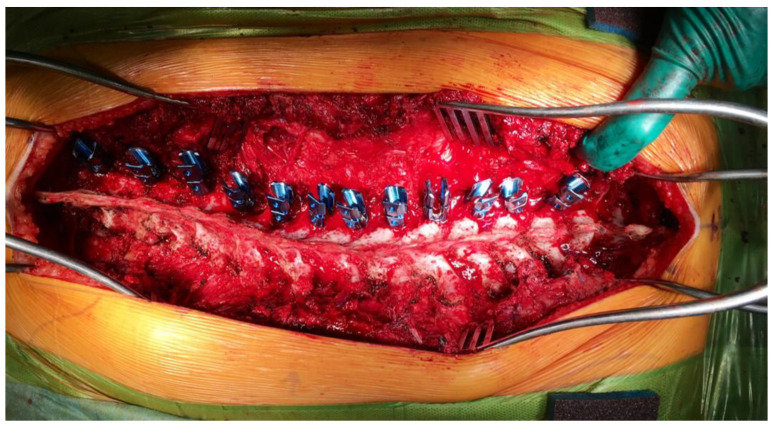
Intraoperative image during posterior spinal fusion surgery for neuromuscular scoliosis, illustrating the placement of pedicle screws on the left side of the spine in full density. The image demonstrates the extensive surgical exposure and the vascular nature of the operative field, highlighting the potential for substantial intraoperative blood loss. The photo emphasizes the need for meticulous hemostasis, targeted antifibrinolytic therapy, and real-time coagulation monitoring to minimize transfusion requirements and perioperative complications.

Surgical correction of spinal deformities in children is frequently associated with substantial blood loss, posing a serious and potentially life-threatening risk (Figure 4). Massive hemorrhage considerably increases perioperative morbidity and mortality in this vulnerable population [55]. Even in patients without preexisting coagulopathy, extensive procedures may lead to acquired consumptive and dilutional coagulopathy, with hypofibrinogenemia often representing the earliest and most critical abnormality. Efforts to maintain normovolemia using crystalloids and red blood cell (RBC) transfusions can further aggravate coagulopathy by diluting clotting factors, underscoring the need for prompt and accurate assessment of hemostasis [27,55].

Goal-directed coagulation management with viscoelastic testing (Rotational Thromboelastometry [ROTEM] or thromboelastography [TEG]) is recommended because it allows timely and targeted replacement therapy (e.g., fibrinogen concentrate or cryoprecipitate when indicated) and provides a more physiological alternative to fixed-ratio transfusion protocols [55]. Given the risks associated with allogeneic transfusions, clearly defined restrictive transfusion thresholds are essential; current pediatric guidelines support thresholds of approximately 7 g/dL for hemodynamically stable patients, individualized to physiology, comorbidity, and surgical complexity [56].

Modern pediatric blood-sparing strategies integrate preoperative anemia optimization, blood-sparing anesthetic techniques, antifibrinolysis, intraoperative cell salvage, and institutional readiness for a massive transfusion protocol (MTP) in high-risk cases. Among these, antifibrinolytics—particularly TXA—are the most effective pharmacologic adjuncts in reducing blood loss during scoliosis surgery. Pediatric dosing regimens vary, including low-dose continuous infusions (e.g., 10–20 mg/kg loading dose followed by 0.25–2 mg/kg/h) and high-dose protocols (e.g., 50 mg/kg loading dose followed by 5 mg/kg/h), selected according to anticipated blood loss, comorbidity, and institutional experience [57].

Comparative pediatric data demonstrate lower estimated blood loss and transfusion requirements with high-dose versus low-dose TXA in AIS [58]. Moreover, disease-specific evidence in NMS confirms the benefit of antifibrinolysis: in cerebral palsy, TXA significantly reduced intraoperative blood loss and cell-salvage transfusion and outperformed ε-aminocaproic acid (EACA), with no increase in adverse events; differences in total allogeneic transfusion were not significant [58]; in spinal muscular atrophy, a 20-year cohort showed a marked reduction in blood loss and transfusion volume with TXA, along with a trend toward fewer postoperative pulmonary complications [59].

Collectively, these findings support the implementation of a standardized TXA protocol as part of a multimodal, goal-directed blood management strategy. In patients with severe, rigid neuromuscular curves or anticipated massive blood loss, many pediatric spine centers adopt higher dosing regimens (e.g., 50–100 mg/kg loading followed by 5–10 mg/kg/h infusion), consistent with institutional guidelines. While current evidence supports the efficacy and safety of TXA in this population, vigilant thromboembolic and neurologic monitoring remains essential [57,58,59].

## 6. Perioperative Complications

The choice of surgical approach for spinal deformity correction—anterior, posterior, or combined—depends on deformity type and severity. Combined approaches are frequently employed for complex and rigid curvatures. These procedures are among the most challenging in spinal surgery and carry the risk of various complications. Reported complication rates for deformity correction and spinal fusion in adolescent idiopathic scoliosis range from 5% to 23% [60]. Higher complication rates are observed in congenital and neuromuscular scoliosis due to associated comorbidities and more complex deformities.

Complications can be classified as intraoperative or postoperative. The most serious involve neurological injuries that may occur during or after surgery. Local intraoperative complications include dural sac injury, bleeding, bone structural damage, and direct organ injury, and those related to improper positioning. Postoperative complications may also include gastrointestinal issues such as ileus and, rarely, pancreatitis.

### 6.1. Surgical Site and Neurological Complications

Postoperative complications include surgical site (SSI) infections, venous thromboembolism, gastrointestinal issues, and implant-related problems. SSI is among the most common complications of spinal surgery, with a reported incidence of approximately 2.7% within 90 days postoperatively [61]. Risk factors include obesity, male sex, prolonged surgery, and neuromuscular disease. Major blood loss requiring transfusion—previously considered routine—is now recognized as a preventable complication.

Anterior spinal artery syndrome (ASAS) represents a severe ischemic insult to the spinal cord and may cause permanent neurological deficits. It results from ischemia in the territory of the anterior spinal artery, which supplies approximately two-thirds of the cord. The mid-thoracic region (T3–T8) is particularly vulnerable due to its relatively poor vascularization, whereas the artery of Adamkiewicz—functionally the most important radicular vessel—enters between T9 and T12 in about 75% of individuals. Mechanical injury, compression, or traction on these arteries can result in profound neurological impairment manifested by motor deficits, sensory disturbances, radicular pain, and bowel or bladder dysfunction.

The prognosis of ASAS remains generally poor, with a mortality rate near 20%, and roughly half of survivors experiencing minimal neurological recovery [62]. Although complete prevention of spinal cord ischemia is impossible, its risk can be mitigated by maintaining adequate mean arterial pressure, avoiding rapid deformity correction, ensuring effective hemostasis, and optimizing oxygen delivery. IONM should be standard practice, combining SSEPs and MEPs where possible, although these modalities may not detect all ischemic events; postoperative deterioration can still occur despite normal intraoperative traces.

### 6.2. Gastrointestinal Complications

Superior mesenteric artery syndrome (SMAS) is a rare but serious gastrointestinal complication caused by duodenal compression between the abdominal aorta and the superior mesenteric artery. First described by Rokitansky in 1842 [63], SMAS is associated with marked weight loss, anorexia nervosa, malabsorption, and hypercatabolic states. Risk factors include asthenic body habitus and previous spinal surgery. Symptoms typically manifest between postoperative days 6–12 as spinal “lengthening” narrows the aortomesenteric angle. Reported prevalence after scoliosis surgery ranges from 0.5% to 2.4% [64,65]. Clinical features include early satiety, nausea, vomiting, post-prandial pain, bloating, and, in severe cases, high intestinal obstruction.

### 6.3. Hemorrhagic Complications

Massive bleeding is defined as blood loss exceeding one total blood volume within 24 h, 50% within 3 h, or ≈10% every 10 min. Scoliosis correction is a prolonged procedure with potential for significant blood loss and acquired coagulopathy. Typical intraoperative blood loss in AIS is often reported at ≈300–1000 mL, varying with curve magnitude, osteotomies, and fusion length; losses are generally higher in NMS.

Blood loss correlates with comorbidities, Cobb angle, scoliosis type, number of fused levels, and surgical duration. Patients with neuromuscular disorders face the highest risk because of severe, rigid deformities and longer operative times. The greatest intraoperative blood loss has been observed in DMD, with substantial losses also observed in myelomeningocele, SMA, and CP [66]. When blood loss exceeds 50% of the estimated circulating volume, the risk of complications rises sharply.

Intraoperative blood management—including goal-directed coagulation therapy, viscoelastic monitoring, and standardized antifibrinolysis with TXA—is detailed in Section 5.5.

### 6.4. Pulmonary Complications

Pulmonary complications are a major cause of postoperative morbidity and mortality in pediatric scoliosis surgery. Their incidence is fivefold higher in children with non-idiopathic scoliosis than in idiopathic cases and greater after anterior than posterior approaches [67]. Common events include atelectasis, hemothorax, pneumothorax, and pleural effusion; less frequent are pneumonia, pulmonary edema, and upper airway obstruction. Children with neuromuscular disorders frequently require postoperative ventilatory support.

In a single-center cohort of 133 children, Al-Iede et al. [67] reported postoperative pulmonary complications in approximately 18% of pediatric patients. Major risk factors included neuromuscular disease, Cobb angle > 90°, reduced forced vital capacity, and elevated serum bicarbonate. Preoperative polysomnography helps identify nocturnal hypoventilation, and preoperative NIV use is associated with significantly fewer postoperative pulmonary complications and an approximately 6-day shorter hospital stay.

### 6.5. Summary and Risk Stratification

The incidence and spectrum of perioperative complications vary according to etiology, deformity severity, and comorbidity (Table 3). NMS carries the highest overall risk, reflecting underlying respiratory compromise, poor nutrition, and prolonged, high-volume fusions. A structured institutional pathway that integrates standardized antifibrinolysis, goal-directed hemostasis, infection prevention, and early respiratory support is essential to reduce morbidity.

## 7. Postoperative Care and Complication Management

The postoperative phase following pediatric scoliosis surgery is critical and requires close monitoring and multidisciplinary coordination to prevent complications and promote recovery. Effective pain control, respiratory support, hemodynamic stability, and early mobilization are cornerstones of postoperative management.

### 7.1. Pain Management

Optimal pain control is best achieved using multimodal analgesia, including scheduled administration of acetaminophen and non-steroidal anti-inflammatory drugs (NSAIDs), supplemented with opioids as needed. Opioid-sparing strategies are particularly important in patients at high risk of respiratory depression or prolonged ventilator weaning. Adjuvants such as gabapentinoids or dexmedetomidine infusions may reduce opioid consumption.

Historically, patient-controlled analgesia with opioids has been the mainstay of postoperative pain management after correction of adolescent idiopathic scoliosis [76]. However, concerns regarding adverse effects, current clinical practice increasingly favors a multimodal analgesic approach that allows early opioid tapering [77]. Epidural catheters delivering continuous local anesthetic infusions can substantially improve postoperative pain control in children undergoing scoliosis surgery [78].

Regional anesthesia techniques, such as bilateral two-level erector spinae plane blocks (ESPBs) at T4 and T10 using 0.2% ropivacaine under ultrasound guidance, may also be considered [79]. This approach does not interfere with intraoperative neuromonitoring and has been shown to reduce intraoperative propofol and remifentanil requirements, as well as the need for postoperative systemic analgesics [80].

Emerging evidence indicates that surgical-site infiltration with liposomal bupivacaine can extend early analgesia and reduce opioid requirements after posterior spinal fusion in adolescents [81]. While evidence in pediatric populations remains limited, its inclusion in multimodal regimens is increasingly explored within ERAS-based protocols [82].

Pain management should therefore combine scheduled NSAIDs with supplemental opioids, local or regional anesthesia, and adjuvant agents, tailored to the child’s clinical condition and risk profile.

### 7.2. Respiratory and Hemodynamic Support

Respiratory support is essential, particularly in patients with restrictive lung disease or impaired cough due to neuromuscular weakness. NIV, chest physiotherapy (including assisted cough), and early mobilization should be implemented proactively. Oxygen supplementation and blood gas monitoring can guide respiratory support during the immediate postoperative period.

Extubation should be carefully planned once the child demonstrates adequate spontaneous ventilation, an effective cough, and stable gas exchange (pH ≥ 7.30 with PaCO_2_ close to the child’s pre-operative/baseline value and SpO_2_ ≥ 92–94% on FiO_2_ ≤ 0.4). In neuromuscular scoliosis or in patients with pre-existing nocturnal hypoventilation, planned post-extubation NIV (e.g., BiPAP) reduces respiratory failure and re-intubation rates [17].

Hemodynamic monitoring should continue in the ICU, particularly for patients with substantial intraoperative blood loss, fluid shifts, or underlying cardiac disease. Regular assessment of hemoglobin levels, coagulation profiles, and fluid balance is essential for goal-directed therapy.

### 7.3. Nutrition and Monitoring

Early initiation of enteral nutrition is recommended to support wound healing and recovery. Nutritional status should be closely monitored, particularly in patients with pre-existing malnutrition or feeding difficulties. In severe neuromuscular disease, gastrostomy-assisted enteral feeding may enhance caloric intake and postoperative rehabilitation outcomes [83].

### 7.4. Complication Surveillance

Common postoperative complications include respiratory failure, wound infection, ileus, nausea, vomiting, and neurological deficits. A high index of suspicion and early intervention are crucial. The emergence of new motor or sensory deficits should prompt immediate neuroimaging and neurosurgical consultation. Immediate decompression of spinal canal stenosis and careful spinal cord deliberation can help restore neurological function in cases of progressive or incomplete deficits [84,85].

### 7.5. Enhanced Recovery and Family Involvement

ERAS pathways for pediatric spinal surgery aim to standardize goal-directed perioperative care and have been associated with fewer complications and shorter hospital stay [86,87] (Table 4). For medically fragile neuromuscular phenotypes, key adaptations include:

- A respiratory pathway with planned postoperative NIV, cough-assist devices, early airway clearance, and nocturnal gas-exchange surveillance (e.g., SMA, advanced DMD).
- A cardiac-first plan in DMD/Becker Muscular Dystrophy (BMD), with continuous ECG monitoring or telemetry and cautious fluid targets.
- Agent selection aligned with disease biology and IONM requirements—notably the absolute avoidance of succinylcholine and volatile anesthetics in DMD/BMD, with TIVA as standard.
- Strict latex-free processes for myelomeningocele and other high-risk groups.
- Individualized VTE prophylaxis, prioritizing mechanical measures and aligning low-molecular-weight heparin use with neuraxial management decisions.

Structured family education using a teach-back approach—covering respiratory equipment use, analgesic plan, wound and urinary tract infection red flags, and escalation pathways—enhances adherence, safety, and confidence at discharge [88,89].

Integrating these measures within institutional ERAS pathways fosters a consistent, multidisciplinary approach to postoperative care and may reduce complication rates in this complex population [86,87,88,89].

## 8. Conclusions

The anesthetic treatment of scoliosis presents numerous challenges, primarily due to the condition’s pathophysiology and complexity. General anesthesia for scoliosis correction in children should be provided exclusively by experienced pediatric anesthesiologists in specialized centers equipped for intensive postoperative care and continuous hemodynamic and respiratory monitoring.

Preoperative management requires a meticulous multidisciplinary approach to evaluate the patient’s overall condition and optimize perioperative safety. Spinal deformity correction represents one of the most complex orthopedic procedures, demanding precise anesthetic planning because of its high-risk nature and technical demands. Key intraoperative priorities include selecting anesthetic techniques compatible with intraoperative neurophysiological monitoring, ensuring optimal patient positioning, maintaining protective ventilation strategies, effective blood-loss control, and comprehensive postoperative intensive care planning (Table 5).

According to current neuroanesthesia guidelines, TIVA delivered via TCI with continuous depth-of-anesthesia monitoring remains the technique of choice for optimizing transcranial motor evoked potentials during spine surgery. Blood-loss management requires vigilant monitoring, timely transfusion decision-making, and targeted hemostatic intervention to maintain hemodynamic stability and prevent coagulopathy.

The implementation of ERAS protocols—which integrate well-defined preoperative, intraoperative, and postoperative elements—has significantly improved perioperative outcomes in pediatric scoliosis surgery (Table 6). Such structured pathways shorten hospitalization, reduce complication rates by up to 63%, attenuate postoperative pain, and may contribute to lower healthcare costs [86,87].

Anesthesia for children with neuromuscular diseases, who frequently present with scoliosis, requires expertise and familiarity with the unique perioperative challenges of this vulnerable patient population [89].

## Figures and Tables

**Table 1:** High-impact anesthetic differences across NMS conditions (DMD/BMD = Duchenne/Becker; SMA = Spinal muscular atrophy; CP = Cerebral palsy; MMC = Myelomeningocele/spina bifida).

Domain	DMD/BMD	SMA	CP	MMC
Airway & aspiration	Macroglossia, OSA; late aspiration risk.	Bulbar dysfunction; high aspiration risk.	Drooling, GERD; secretions.	GERD common; postsurgical airway changes possible.
Ventilation	Restrictive disease; plan postop NIV, cough-assist.	Severe respiratory insufficiency; NIV training, cough-assist.	Variable capacity; atelectasis-prone—gentle strategy.	Prone abdomen may impair venous return.
Cardiac	Dilated cardiomyopathy, arrhythmias → echo/ECG.	No primary cardiomyopathy; respiratory load fatigue.	Occasional congenital defects; PH if chronic hypoventilation.	Structural defects possible; preload-sensitive in prone.
Anesthetic agents	Absolute: no succinylcholine, no volatile → TIVA.	Prefer TIVA with IONM; volatile only if necessary.	Either approach; watch antiepileptic interactions.	Latex-free OR; agents per IONM/hemodynamics.
Neuromuscular blockers	Sux contraindicated; ↑ sensitivity to NDNMB; use TOF, consider sugammadex.	↑ sensitivity to NDNMB; minimal dosing; TOF; sugammadex.	Spasticity may confound TOF; individualize dosing.	Prior neurosurgery; anatomic variation—use TOF.
IONM strategy	TIVA mandatory, MEP/SSEP preserved.	Prefer TIVA with IONM; minimize volatiles.	Either; with IONM prefer TIVA.	Either; with IONM prefer TIVA.
Perioperative therapy interactions	Chronic steroids → consider stress-dose; cardiac meds.	Nusinersen/risdiplam do not change anesthesia plan; prioritize respiratory support.	Continue antiepileptics; check baclofen pump.	Latex allergy risk very high → strict latex-free setup.

Abbreviations: OSA, obstructive sleep apnea; GERD, gastroesophageal reflux disease; NIV, noninvasive ventilation; PH, pulmonary hypertension; IONM, intraoperative neurophysiological monitoring; TIVA, total intravenous anesthesia; NDNMB, non-depolarizing neuromuscular blocker; TOF, train-of-four; MEP, motor evoked potential; SSEP, somatosensory evoked potential; OR, operating room; echo, echocardiography; ECG, electrocardiography.

**Table 2:** Multidisciplinary checklist for immediate interventions following IONM signal loss.

Neurophysiologist/IONM Specialist	Anesthesiologist	Surgeon
Repeat IONM test.	Ensure no interfering anesthetic agents are being administered.	Assess any surgical intervention immediately preceding the IONM alert.
Verify electrode placement and impedance; optimize stimulation parameters and IONM settings.	Maintain mean arterial pressure (MAP) ≥ 70 mmHg or 10–20% above preoperative baseline.	Consider temporarily halting the procedure; observe for recovery of IONM signals.
Eliminate artifacts and electrical noise.	Reassess head and limb positioning, particularly in cases of unilateral signal loss.	If necessary, ensure intraoperative imaging is available for further assessment.
Evaluate potential waveform changes and onset timing.	Adjust hematocrit (target Hct > 30%), correct pH and pCO_2_, maintain normothermia and normoglycemia.	Assess signal recovery post-intervention. Be prepared for a wake-up test or to modify the surgical strategy.

Abbreviations: IONM, intraoperative neurophysiological monitoring; MAP, mean arterial pressure; Hct, hematocrit.

**Table 3:** Major perioperative complications in pediatric scoliosis surgery.

Type of Complication	Typical Incidence	Main Risk Factors	Preventive/Mitigating Strategies
Neurological injury	AIS: ~0.3–1% [68,69]; NMS: higher than AIS (heterogeneous cohorts) [70]	Severe curve > 90°, rapid correction, hypotension	Maintain MAP ≥ 65 mmHg; combined SSEP/MEP IONM; avoid overly rapid correction
Surgical site infection (SSI)	~2.7% within 90 days after posterior fusion for AIS [61] (higher ranges reported across pediatric spinal fusion)	NMS, obesity, male sex, prolonged surgery	Antibiotic prophylaxis; normothermia; meticulous wound care
Hemorrhage > 50% EBV	Incidence varies by etiology and fusion length; highest in DMD, followed by MMC, SMA, and CP [66]	Long, rigid fusion; NMS; high Cobb angle; prolonged operative time	TXA protocol; cell salvage; viscoelastic-guided hemostasis (see Section 5.4)
Pulmonary complications	~18% overall in pediatric scoliosis surgery; higher in non-idiopathic vs. AIS; higher after anterior approach [67,71,72,73]	NMS, Cobb > 90°, low FVC, ↑HCO_3_^−^	Preoperative polysomnography; prophylactic NIV; lung-protective ventilation; early extubation
Superior mesenteric artery syndrome (SMAS)	0.5–2.4% after scoliosis correction [64,65]	Low BMI/asthenic habitus; rapid correction; prior spinal surgery	Nutritional optimization; gradual correction; early feeding protocol
Venous thromboembolism (VTE)	<1% in pediatric orthopedics; pharmacologic thromboprophylaxis is not routine—individualize by risk [74,75]	Prolonged immobility, CVC, obesity, trauma	Mechanical prophylaxis; selective LMWH per risk (see Section 3: Preoperative Preparation)

Abbreviations: AIS—adolescent idiopathic scoliosis; NMS—neuromuscular scoliosis; EBV—estimated blood volume; DMD—Duchenne muscular dystrophy; MMC—myelomeningocele; CP—cerebral palsy; MAP—mean arterial pressure; IONM—intraoperative neuromonitoring; SSEP—somatosensory evoked potentials; MEP—motor evoked potentials; FVC—forced vital capacity; NIV—non-invasive ventilation; CVC—central venous catheter; TXA—tranexamic acid; LMWH—low-molecular-weight heparin.

**Table 4:** Overview of key ERAS principles in pediatric scoliosis surgery (adapted from [86,87,88,89]).

ERAS Domain	Core Components	Specific Adaptations for Neuromuscular Scoliosis (NMS)
Preoperative optimization	Multidisciplinary evaluation (anesthesiology, neurology, pulmonology, cardiology, nutrition, physiotherapy); patient and family education; anemia management; antifibrinolytic planning	Prehabilitation—Cough training; airway clearance; PEG feeding if malnourished; latex avoidance; individualized anesthesia plan.
Anesthetic management	IONM-compatible TIVA (propofol/remifentanil); lung-protective ventilation; temperature and fluid management; antifibrinolytic therapy (TXA); PONV prophylaxis; intubation strategy compatible with IONM (short-acting NMB for intubation only, then avoid)	Anesthesia (DMD/BMD)—TIVA mandatory; strict avoidance of succinylcholine and volatile agents; real-time IONM coordination.
Intraoperative care	Normothermia; goal-directed fluid therapy; viscoelastic-guided hemostasis; restrictive transfusion thresholds.	Higher vigilance for coagulopathy; maintain age-appropriate MAP targets (near baseline; avoid hypotension); minimize mechanical stress during deformity correction
Postoperative pain control	Scheduled acetaminophen ± NSAIDs (if not contraindicated); adjuncts (gabapentinoids, low-dose ketamine or dexmedetomidine); regional techniques (ESPB/parasagittal blocks, wound infiltration); early mobilization.	Prefer regional/wound infiltration over neuraxial if neuro exam or respiratory risk; avoid routine epidural; consider liposomal bupivacaine (limited evidence); no basal opioid (PCA demand-only); continuous oximetry ± capnography, planned nocturnal NIV; monitor for respiratory depression.
Respiratory and cardiovascular support	Incentive spirometry; chest physiotherapy; early ambulation; hemodynamic stability monitoring	Planned extubation with immediate NIV (BiPAP); nocturnal gas-exchange monitoring; telemetry in cardiac involvement
Nutrition and early recovery	Early enteral feeding; avoidance of prolonged fasting; prevention of ileus and nausea	PEG or nasogastric feeding for undernourished or dysphagic children; nutritional supplementation
Family involvement	Teach-back education on airway equipment, pain plan, wound/infection red flags (incl. UTI), and a clear escalation protocol.	Extended caregiver training and participation in daily respiratory care; coordination with home-ventilation teams

Abbreviations: ERAS—enhanced recovery after surgery; NMS—neuromuscular scoliosis; DMD—Duchenne muscular dystrophy; BMD—Becker muscular dystrophy; PEG—percutaneous endoscopic gastrostomy; TXA—tranexamic acid; TIVA—total intravenous anesthesia; IONM—intraoperative neuromonitoring; NIV—non-invasive ventilation; MAP—mean arterial pressure; ESPB—erector spinae plane block; UTI—urinary tract infection.

**Table 5:** Perioperative management in children undergoing scoliosis surgery (NMS-adapted).

Preoperative Preparation	Intraoperative Management	Postoperative Management
• Determine scoliosis type/etiology and age at onset.	• TIVA with TCI (propofol/remifentanil) and continuous depth monitoring (DMD/BMD: no volatile).	• Early extubation in OR when feasible (plan NIV/cough-assist if SMA/advanced DMD).
• Focused history: pulmonary function (cough effectiveness, obstruction); palpitations/syncope (esp. DMD/BMD).	• IV induction of anesthesia.	• Multimodal analgesia; antiemetic prophylaxis as indicated.
• Targeted exam: airway & cervical mobility, neurologic status, pulmonary, cardiac assessment (echo/ECG if DMD/BMD).	• Non-depolarizing NMB for intubation; avoid succinylcholine (DMD/BMD).	• Ensure adequate ventilation/oxygenation; lung-protective strategy if restrictive mechanics.
• Labs and type & screen/cross-match; plan blood products by anticipated loss.	• Prepare for difficult airway (bulbar SMA/advanced CP; adjuncts/backup).	• Anticipate postoperative mechanical ventilation based on—Preop: etiology, Cobb angle, spirometry (FEV_1_%, FVC%), comorbidities—Intraop: levels fused, instability/course—Modifiable: transfusion burden, hypothermia
• Optimization: nutrition, hydration, infection screen; ERAS briefing (latex-free plan if MMC).	• Advanced monitoring: invasive arterial pressure; consider central venous access if vasoactives likely; hourly urine output; core temperature.	• ICU/step-down as indicated; early physiotherapy and cough-assist/NIV where appropriate.
• Vascular access plan (US-guided if difficult); anesthesia/analgesia plan aligned with IONM if used.	• Secure reliable IV access (≥2 large-bore peripherals; ultrasound guidance as needed).	• Catheter care (PIV/CVC/urinary) with early removal when safe; early enteral nutrition.
	• Prone positioning with chest/abdominal support; protect pressure points/eyes (fragile skin; MMC: strict latex-free OR).	• VTE prevention: mechanical for all; pharmacologic selectively per risk and neuraxial timing.
	• Blood-loss management: cell salvage; minimize allogeneic transfusion; antifibrinolytics; viscoelastic testing to guide therapy.	• Discharge education (analgesics schedule, wound care, red flags, respiratory device use/alarms).

Abbreviations: NMS = neuromuscular scoliosis; DMD/BMD = Duchenne/Becker muscular dystrophy; SMA = spinal muscular atrophy; CP = cerebral palsy; MMC = myelomeningocele/spina bifida; TIVA = total intravenous anesthesia; TCI = target-controlled infusion; IONM = intraoperative neurophysiological monitoring; NMB = neuromuscular blocker; US = ultrasound; PIV = peripheral IV; CVC = central venous catheter; NIV = non-invasive ventilation; VTE = venous thromboembolism; OR = operating room. Notes (NMS-specific flags): DMD/BMD → no succinylcholine, no volatile (TIVA standard); SMA/advanced DMD → planned NIV/cough-assist; MMC → strict latex-free (OR and disposables), UTI surveillance; CP → continue antiepileptics and check baclofen pump.

**Table 6:** ERAS in scoliosis surgery: core elements and NMS-specific adaptations.

Phase	Core ERAS Elements (Apply to All)	NMS-Specific Adaptations (High-Impact Nuances)
Preoperative	Patient/parent education; premedication; fasting; nutritional assessment.	Respiratory stratification with planned NIV and cough-assist (SMA/advanced DMD); cardiac-first planning in DMD/BMD (continuous ECG monitoring as indicated, cautious fluids); agent selection briefing (no succinylcholine/volatile in DMD/BMD → TIVA); latex-free pathway for MMC; continue antiepileptics (CP), check baclofen pump; aspiration mitigation in bulbar phenotypes.
Intraoperative	Goal-directed fluids; opioid-sparing anesthesia; temperature monitoring/normothermia; antibiotic prophylaxis; meticulous hemostasis; IONM-compatible technique.	TIVA with IONM (minimize volatiles; DMD/BMD: TIVA mandatory); lung-protective ventilation (VT ~6–7 mL·kg^−^^1^ PBW, individualized PEEP, gentle recruitment); cautious NDNMB titration with TOF (prefer sugammadex in DMD/SMA); latex-free OR for MMC; enhanced padding/pressure-injury prevention.
Postoperative	ICU/step-down as indicated; fluid optimization; appropriate catheter care; multimodal analgesia; early nutrition; structured discharge education.	Risk-stratified PICU with planned NIV/cough-assist (SMA/advanced DMD); telemetry where cardiomyopathy/arrhythmia risk (DMD/BMD); mechanical VTE prophylaxis for all, pharmacologic selectively and timed vs. neuraxial; UTI surveillance (MMC).

Abbreviations: ERAS—enhanced recovery after surgery; NMS—neuromuscular scoliosis; DMD—Duchenne muscular dystrophy; BMD—Becker muscular dystrophy; SMA—spinal muscular atrophy; CP—cerebral palsy; MMC—myelomeningocele/spina bifida; NIV—non-invasive ventilation; TIVA—total intravenous anesthesia; IONM—intraoperative neurophysiological monitoring; PBW—predicted body weight; PEEP—positive end-expiratory pressure; NDNMB—non-depolarizing neuromuscular blocker; TOF—train-of-four; VTE—venous thromboembolism; UTI—urinary tract infection; OR—operating room; PICU—pediatric intensive care unit.

## Data Availability

Not applicable. No new data were created or analyzed in this study.

## Abbreviations

AIS	Adolescent Idiopathic Scoliosis
BMD	Becker Muscular Dystrophy
CP	Cerebral Palsy
CT	Computed Tomography
CVC	Central Venous Catheter
DMD	Duchenne Muscular Dystrophy
EBV	Estimated Blood Volume
ECG	Electrocardiography
ERAS	Enhanced Recovery After Surgery
ESPB	Erector Spinae Plane Block
FVC	Forced Vital Capacity
GMFCS	Gross Motor Function Classification System
IONM	Intraoperative Neurophysiological Monitoring
IV (PIV)	Peripheral Intravenous (catheter)
LMWH	Low-Molecular-Weight Heparin
MAP	Mean Arterial Pressure
MEP	Motor Evoked Potential
MMC	Myelomeningocele (Spina Bifida)
NDNMB	Non-Depolarizing Neuromuscular Blocker
NIV	Non-Invasive Ventilation
NMB	Neuromuscular Blocker
NMS	Neuromuscular Scoliosis
NSAID	Non-Steroidal Anti-Inflammatory Drug
OR	Operating Room
PBW	Predicted Body Weight
PEEP	Positive End-Expiratory Pressure
PICU	Pediatric Intensive Care Unit
SMA	Spinal Muscular Atrophy
SSEP	Somatosensory Evoked Potential
TCI	Target-Controlled Infusion
TIVA	Total Intravenous Anesthesia
TOF	Train-of-Four
TXA	Tranexamic Acid
UTI	Urinary Tract Infection
VTE	Venous Thromboembolism
VT	Tidal Volume
